# Survivors of Ebola Virus Disease Develop Polyfunctional Antibody Responses

**DOI:** 10.1093/infdis/jiz364

**Published:** 2019-07-12

**Authors:** Bronwyn M Gunn, Vicky Roy, Marcus M Karim, Jessica N Hartnett, Todd J Suscovich, Augustine Goba, Mambu Momoh, John Demby Sandi, Lansana Kanneh, Kristian G Andersen, Jeffrey G Shaffer, John S Schieffelin, Robert F Garry, Donald S Grant, Galit Alter

**Affiliations:** 1 Ragon Institute of Massachusetts General Hospital, Massachusetts Institute of Technology, and Harvard, Cambridge, Massachusetts; 2 Department of Microbiology and Immunology, Tulane University School of Medicine, New Orleans, Louisiana; 3 Viral Hemorrhagic Fever Program, Kenema Government Hospital; 4 Eastern Polytechnic University, Kenema, Sierra Leone; 5 Department of Immunology and Microbiology, Scripps Research Institute; 6 Scripps Research Translational Institute, La Jolla, California; 7 Department of Biostatistics and Bioinformatics, Tulane School of Public Health and Tropical Medicine, New Orleans, Louisiana; 8 Ministry of Health and Sanitation, Freetown, Sierra Leone

**Keywords:** Ebola virus, antibody, innate immune effector function

## Abstract

Monoclonal antibodies can mediate protection against Ebola virus (EBOV) infection through direct neutralization as well as through the recruitment of innate immune effector functions. However, the antibody functional response following survival of acute EBOV disease has not been well characterized. In this study, serum antibodies from Ebola virus disease (EVD) survivors from Sierra Leone were profiled to capture variation in overall subclass/isotype abundance, neutralizing activity, and innate immune effector functions. Antibodies from EVD survivors exhibited robust innate immune effector functions, mediated primarily by IgG1 and IgA1. In conclusion, development of functional antibodies follows survival of acute EVD.

## Teaser

The humoral immune response in human survivors of Ebola virus disease includes the development of neutralizing antibodies and polyfunctional IgG1 and IgA antibodies that can mediate effector functions against the Ebola virus glycoprotein via multiple innate immune effector cell types.

The 2013–2016 Ebola virus disease (EVD) epidemic in West Africa and current outbreaks in the Democratic Republic of Congo indicate that highly pathogenic filoviruses remain a significant threat to human health. The ability of antibodies to provide protection from a lethal Ebola virus (EBOV) challenge has been demonstrated in the context of pre- and postexposure administration of EBOV glycoprotein (GP)–specific monoclonal antibodies (mAbs) [1]. Antibodies can provide antiviral protection through direct restriction of viral entry via the antigen binding domain (Fab)–mediated neutralization and through recruitment of innate immune effector functions via the antibody constant domain (Fc). While Fab-mediated neutralization is undoubtedly a key component of protection against EBOV, recent studies demonstrate a role for antibody Fc-mediated innate immune effector functions, such as phagocytosis and natural killer (NK) cell activation, in mAb-mediated protection against EBOV [2–4].

EVD survivors develop both humoral and cellular immunity against several EBOV proteins, including GP, secreted GP (sGP), nucleoprotein (NP), and matrix protein VP40 [5–7]. Early studies indicated that development of an antibody response was associated with survival from EVD [5], and while neutralizing antibodies develop in EVD survivors [8], the ability of EVD-induced antibodies to mediate innate immune effector functions has not been evaluated. Analysis of therapeutic antibodies in animal models demonstrated that antibodies with ability to neutralize and recruit multiple effector functions provided protection against Ebola virus, and thus we hypothesized that similar antibody profiles develop in EVD survivors. Here, we profiled antibodies from a small cohort of EVD survivors from Kenema, Sierra Leone, for the ability to induce innate immune effector functions. A majority of survivors develop both neutralizing antibodies and polyfunctional antibodies that induce multiple innate immune cell functions, linked to higher levels of EBOV-specific immunoglobulin G1 (IgG1) and immunoglobulin A (IgA) antibodies. Our data suggest that development of neutralizing antibodies and polyfunctional IgG1 and IgA antibodies are natural components of humoral immunity following survival of acute EVD.

## METHODS

### Subjects

Plasma samples were collected from individuals with documented clinical history of EVD approximately 6 months following survival (Supplementary Table 1), household contacts, or EBOV GP-seronegative individuals. EVD patients received standard of care at Kenema Government Hospital (eg, intravenous fluid replacement and administration of antibiotics as determined by the treating physician). Peripheral blood mononuclear cells were collected from Massachusetts General Hospital (MGH) blood bank donations. All subjects provided written consent. This study was approved by the Institutional Review Board of the Human Subjects Committee of MGH, the Tulane University Institutional Review Board, and the Sierra Leone Ethics and Scientific Review Committee.

### Antigen-Specific Antibody Levels

Recombinant GP, sGP, VP40 (IBT Bioservices), and NP (Sino Biologics) were coupled to MagPlex beads (Luminex) according to previously published protocols [9]. Samples were diluted and incubated with antigen-coupled beads for 2 hours. Following bead washing, antibody subclasses (IgG1, IgG2, IgG3, IgG4) and isotypes (immunoglobulin M [IgM], IgA1, IgA2) were detected using PE-labeled secondary antibodies (0.65 µg/mL; Southern Biotech). The geometric mean fluorescence intensity (gMFI) of 30 beads/region was analyzed on a Flexmap 3D instrument (Luminex). A subclass and antigen-specific cutoff was determined by calculating gMFI and standard deviation of the responses measured in the controls and household contacts (n = 23). The threshold of positivity was defined as gMFI plus twice the standard deviations of control and household contact measurements.

### Phagocytosis

Biotinylated GP was conjugated to yellow-green Neutravidin beads (Thermo Fisher) and incubated with samples for 2 hours prior to adding white blood cells from human donor peripheral blood (5 × 10^4^ cells/well) for 1 hour (antibody-dependent phagocytosis by neutrophils [ADNP]) or adding THP-1 cells at 2.5 × 10^4^ cells/well for 18 hours (antibody-dependent cellular phagocytosis by monocytes [ADCP]). Uptake of antibody-bead complexes by cells was determined by flow cytometry, and a phagocytic score was determined: (% fluorescein isothiocyanate [FITC]^+^ cells) × (gMFI FITC^+^) / 10 000.

### Antibody-Mediated Complement Deposition

Biotinylated GP-coated red Neutravidin beads (Thermo Fisher) were incubated with heat-inactivated samples. Guinea pig complement (Cedarlane Labs) diluted in veronal buffer containing calcium and magnesium (Boston Bioproducts) was incubated with antibody-bead complexes for 20 minutes, and C3 deposition onto beads was detected using an anti–guinea pig C3 antibody (MP Biomedicals) and measured by flow cytometry.

### Antibody-Dependent NK Cell Degranulation

Enzyme-linked immunosorbent assay (ELISA) plates were coated with GP antigen (300 ng/well). Wells were washed, blocked, and incubated with samples for 2 hours prior to adding NK cells enriched from the peripheral blood of human donors (5 × 10^4^ cells/well) for 5 hours with brefeldin A, GolgiStop, and anti-CD107a (BD Biosciences). Intracellular cytokine staining to detect interferon-γ and macrophage inflammatory protein-1β (BD Biosciences) was performed using Fix/Perm (Life Technologies), and cells were analyzed by flow cytometry.

### Neutralizing Activity

Two-fold dilutions of samples (1:12–1:384) were incubated with 4 × 10^4^ renilla luciferase units/well of EBOV GP-pseudotyped vesicular stomatitis virus expressing luciferase (IBT Bioservices), followed by overnight incubation with VeroE6 monolayers. Cells were lysed and luciferase activity was determined using a luciferase-activating reagent (Promega). Endpoint neutralization titers were defined as the last dilution that reduced luciferase activity by 50% or 80% compared to control wells.

### Depletions

Samples were diluted and incubated with CaptureSelect IgG1, IgA, or control resins (Thermo Fisher Scientific) for 1 hour. Depleted samples were collected by centrifugation using a 30–40 µm filter plate. Depletion was confirmed using a multiplexed human isotyping kit (MilliporeSigma).

### Network Analysis

Associations between antibody features were determined using nonparametric Spearman correlation coefficient. Statistically significant associations after Bonferroni correction for multiple comparisons (adjusted *P* < .05) were used to generate networks in Cytoscape (version 3.4.0).

### Statistical Analysis

Univariate analyses were performed using Prism 7 software. Kruskal–Wallis with Dunn multiple correction test or Mann–Whitney with Bonferroni correction was used to determine statistical significance between groups.

## RESULTS

### EVD Survivor Antibody Reactivity Against EBOV Proteins

**Figure 1.**
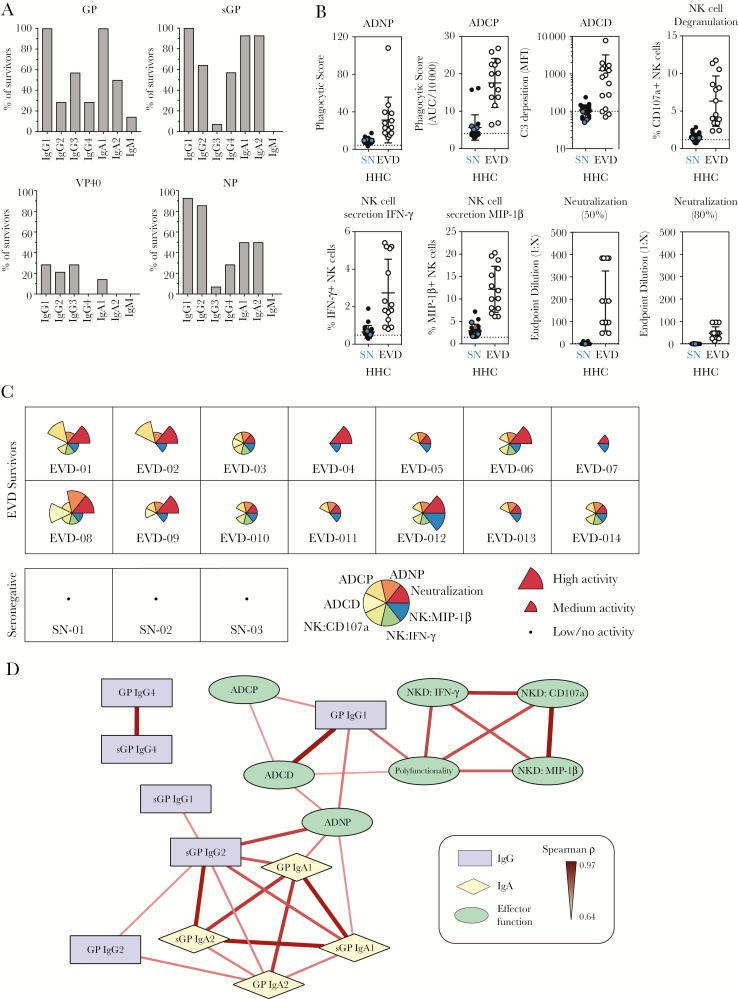
Antibody functional diversity in Ebola virus disease (EVD) survivors. *A*, Percentage of survivors (n = 14) who developed immunoglobulin (Ig) G1, IgG2, IgG3, IgG4, IgA1, IgA2, and IgM responses against the Ebola virus glycoprotein (GP), secreted glycoprotein (sGP), VP40, and nucleoprotein (NP) that were 2 times over the geometric mean fluorescence intensity (MFI) of seronegative (SN) and household contact (HHC) samples (n = 23) from Sierra Leone were calculated. *B*, Induction of the indicated innate immune effector functions (antibody-dependent phagocytosis by neutrophils [ADNP], antibody-dependent phagocytosis by monocytes [ADCP], antibody-dependent complement deposition [ADCD], and natural killer [NK] cell degranulation and activation) and neutralizing activity (endpoint dilution of plasma that achieved 50% or 80% neutralization) by SN samples (blue circles; n = 3), HHC (closed circles; n = 20), or samples from EVD survivors (open circles) were measured. *C*, The magnitude of the functional response for each sample was characterized into high, medium, and low/negligible for each function, based on cutoffs defined by positive and negative controls, and is plotted for each individual. Each wedge is color coded by function, with the size of the wedge corresponding to the magnitude of the response in the respective functional assay. The survivor sample identifier is indicated in bottom of each box. *D*, Correlation network analysis of statistically significant associations (Bonferroni adjusted *P* < .05) between GP-specific functional activity and levels of GP- and sGP-specific antibody levels. Positive correlations between features are indicated by a red connecting line, the strength of correlation is indicated by weight of the connecting line, and the background color of the features indicates the category of the feature (eg, functional activity, IgG, IgA), as indicated in the boxed legend.

We determined relative antibody levels of IgG subclasses and isotypes specific for EBOV GP, sGP, NP, and VP40 of EVD survivors, seronegative individuals, and household contacts. Survivors demonstrated higher antibody responses against GP, sGP, and NP compared to seronegative and household contact controls from the same region across several IgG subclasses and isotypes (Supplementary Figure 1). We observed heterogeneity among the subclasses and isotypes across survivors with regard to antigen specificity. Less than 30% of survivors developed responses against VP40 whereas >90% of the survivors developed responses against GP, sGP, and NP (Figure 1A). Notably, IgG1 and IgA1 responses against GP and sGP were detected in all survivors.

### Induction of Innate Immune Effector Functions by GP-Specific Antibodies

As GP is exposed on virions and infected cells, we measured the ability of GP-specific antibodies to mediate neutralization and induce innate immune effector functions: ADNP, ADCP, ADCD, and antibody-dependent NK cell activation (Figure 1B). The survivor samples induced higher levels of neutralization and functional responses compared to the samples from seronegative individuals and household contacts (Figure 1B), although heterogeneity in responses between EVD survivors was observed. To visualize the antibody profile of each survivor, we classified each response into high, medium, and low/no activity, and generated a profile plot for each sample (Figure 1C). A range of profiles was observed: highly polyfunctional profiles (eg, EVD-008, EVD-003), discrete functional profiles characterized by induction of a subset of innate immune functions (eg, EVD-011, EVD-005), and 2 samples induced little functional activity (EVD-004, EVD-007), linked to low levels of IgG1 (Supplementary Figure 1).

To determine if either neutralizing or effector function activity was associated with a specific antibody subclass and isotype, we performed a network analysis using significant associations between GP- and sGP-specific antibody levels and functional activity induced by the survivor samples (Figure 1D). While neutralizing antibody levels were weakly associated with ADCD and GP-specific IgG1 levels (data not shown), effector function was predominantly associated with GP-specific IgG1 levels. Interestingly, GP- and sGP-specific IgA1 were associated with ADNP activity, pointing to the role of antibody isotypes beyond IgG in mediating effector function. To determine if levels of NP-, VP40-, sGP-, and GP-specific antibody responses were coordinated in survivors, we performed network analysis of statistically significant associations between the antibody levels across antigen specificities (Supplementary Figure 2). We observed a highly interconnected network of antibody responses, with a high degree of coordination of IgG1 and IgA responses across antigen specificities, suggesting that development of IgG1 and IgA responses may be tightly coordinated across antigens in EVD.

### Both IgG1 and IgA Contribute to Functional Activity

**Figure 2.**
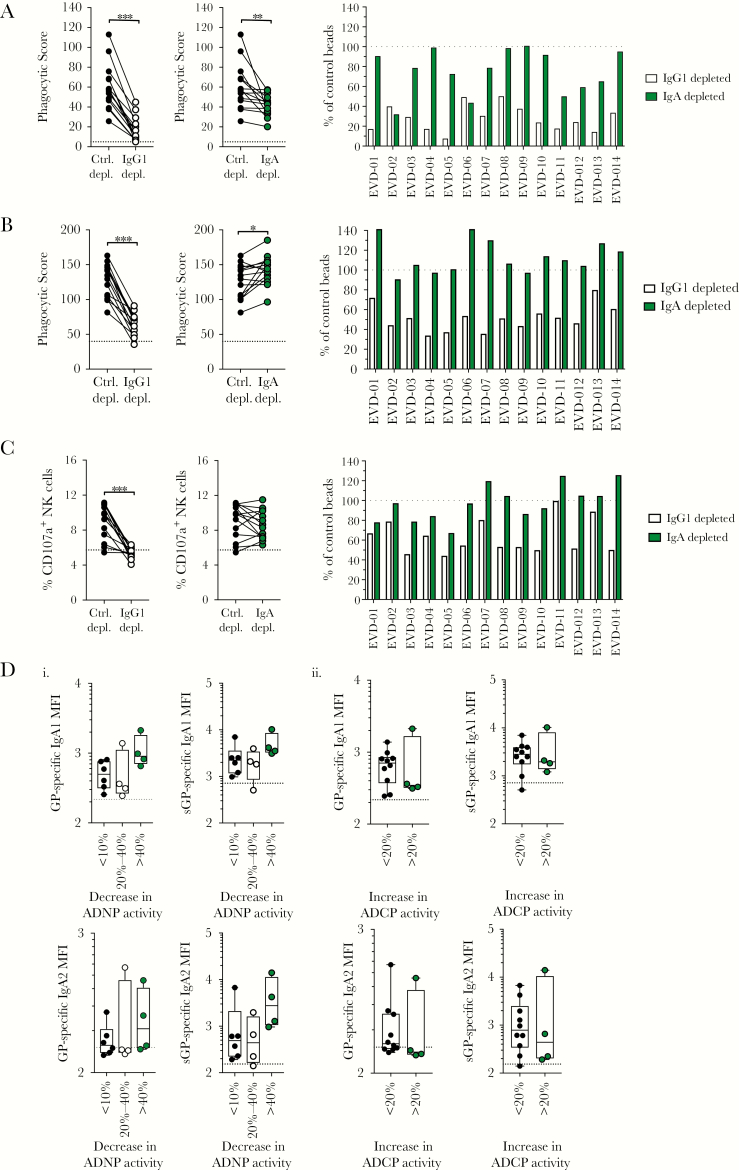
Both immunoglobulin G1 (IgG1) and immunoglobulin A1 (IgA1) mediate innate immune effector function. *A–C*, Samples from Ebola virus disease (EVD) survivors were depleted of either IgG1 (open circles) or IgA (green circles) using resins. Depleted samples were compared to control depletions (irrelevant; closed circles) for induction of antibody-dependent phagocytosis by neutrophils (ADNP; *A*), antibody-dependent phagocytosis by monocytes (ADCP; *B*), or natural killer (NK) cell degranulation (*C*). The raw functional activity scores are shown in the 2 graphs on the left, where the dotted line represents the functional activity of seronegative samples. The functional activity of IgG1-depleted (open bars) or IgA-depleted (green bars) for each survivor is shown in the graph on the right and expressed as the percentage of functional activity compared to control depleted samples, where the dotted line represents 100%. ***P < .001, **P < .01, *P < .05 by Wilcoxon matched-pairs signed-rank test. *D*, Samples were binned into different categories based on reduction of ADNP functional activity (*i*) or increase in ADCP activity (*ii*) following IgA depletion. Samples were binned into 3 categories based on ADNP activity: reduction of <10% (closed circles), 20%–40% (open circles), and >40% (green circles). Samples were binned into 2 categories based on ADCP activity: an increase of <20% or >20%. Levels of glycoprotein (GP)– and secreted glycoprotein (sGP)–specific IgA1 (top row) or IgA2 (bottom) (mean fluorescence intensity [MFI]) were compared between the groups by Kruskal–Wallis test (ADNP) or Mann–Whitney test (ADCP).

Given the coordination of IgG1 and IgA responses and association with induction of innate immune effector functions, we hypothesized that IgG1 and IgA may mediate disparate functional activities. We depleted IgG1 and IgA from the samples using resins and evaluated depleted samples for induction of effector functions from neutrophils, monocytes, and NK cells. Depletion of IgG1 from the samples significantly reduced the ability of all samples to mediate functional activity compared to samples incubated with a control bead resin (Figure 2A–C), indicating that IgG1 antibodies are the primary drivers of innate immune effector function.

Depletion of IgA had a more heterogenous impact on functional activity. ADNP activity was reduced in half of the IgA-depleted samples, and 4 of the samples had reductions of ADNP activity of >40% (Figure 2A). Unexpectedly, depletion of IgA in 4 of the 14 samples resulted in an increase in ADCP activity of >20%, suggesting that IgA may interfere or block ADCP activity (Figure 2B). No significant impact of IgA depletion on NK cell degranulation was observed (Figure 2C), consistent with the lack of CD89 on NK cells.

As IgA1 and IgA2 may have differential impact on antiviral immunity [10], we next determined if the levels of virus-specific IgA1 and IgA2 could account for the differential impact of IgA on ADNP and ADCP activity. We binned samples into those where depletion of IgA resulted in <10%, 20%–40%, or >40% reduction of ADNP activity (Figure 2D-i). Although no significant differences in IgA1 and IgA2 levels were observed between groups, samples with >40% reduction of activity trended toward higher GP-specific IgA1 and higher sGP-specific IgA1 and IgA2 levels. As IgA1 levels were highest in samples with >40% reduction in activity, IgA1 likely contributes to ADNP activity. However, as sGP-specific IgA2 levels are also elevated in those samples, both sGP-specific IgA subclasses may drive ADNP activity. To determine if IgA1/IgA2 levels was associated with enhanced ADCP activity following IgA depletion, samples were binned into those with ADCP activity >20% or <20%. No difference in levels of GP- or sGP-specific IgA1 or IgA2 were observed between groups (Figure 2D-ii), suggesting that titer alone cannot account for the differences in IgA-mediated inhibition of ADCP activity.

## DISCUSSION

Studies in animal models indicates that protective antibodies use both neutralization and recruitment of innate immune effector functions to provide protection against a lethal EBOV infection. Here we show that the humoral immune response that develops in human survivors of EVD resembles that of protective monoclonal antibodies, marked by the development of both neutralizing and polyfunctional antibodies. Importantly, our analysis demonstrates that both IgG1 and IgA antibodies mediate effector functions.

We found that all survivors developed antibody responses against GP and sGP, consistent with previous reports [5, 11]. Recent analysis of CD8^+^ T-cell responses in the same EVD survivors evaluated here demonstrated a relatively low abundance of GP-specific CD8^+^ T cells, yet much higher levels of NP- and VP40-specific CD8^+^ T cells [6]. The split between development of GP-specific antibodies and development of NP- and VP40-specific CD8^+^ T cells may indicate that the two branches of adaptive immunity are differentially shaped by distinct EBOV proteins that may complement each other to maximize immunity.

Given the mucosal nature of the initial infection, the development functional IgA against GP may provide protection at mucosal sites. Macrophage and neutrophils within the mucosa express high levels of FcαR [12], and thus, IgA-mediated induction of phagocytosis may contribute to rapid control/clearance of virus/infected cells at the mucosa. Finally, the humoral response induced by the protective vaccine candidate recombinant vesicular stomatitis virus–EBOV includes induction of GP-specific IgA [13], and thus EBOV-specific IgA may be a critical component of a protective response against EBOV.

## Supplementary Data

Supplementary materials are available at *The Journal of Infectious Diseases* online. Consisting of data provided by the authors to benefit the reader, the posted materials are not copyedited and are the sole responsibility of the authors, so questions or comments should be addressed to the corresponding author.

## Supplementary Material

jiz364_suppl_Supplementary_Figure_S1

jiz364_suppl_Supplementary_Figure_S2

jiz364_suppl_Supplementary_Legends

jiz364_suppl_Supplementary_Table_S1

## Notes

***Acknowledgments.*** We thank the participants of this study, members of the Alter laboratory, and the Center for Viral Systems Biology for discussion and support, especially Laura Hughes. We thank Simberie Jalloh, Christopher Bishop, Tynette Hills, and Allyson Haislip for logistical support.

***Financial support.*** This work was supported by funding from the National Institute of Allergy and Infectious Diseases (grant numbers U19 AI35995 and R01 AI123535).

***Potential conflicts of interest.*** All authors: No reported conflicts of interest. All authors have submitted the ICMJE Form for Disclosure of Potential Conflicts of Interest. Conflicts that the editors consider relevant to the content of the manuscript have been disclosed.

## References

[1] Wilson JA, Hevey M, Bakken R, et al. Epitopes involved in antibody-mediated protection from Ebola virus. Science 2000; 287:1664–6. 10698744 10.1126/science.287.5458.1664

[2] Gunn BM, Yu WH, Karim MM, et al. A role for Fc function in therapeutic monoclonal antibody-mediated protection against Ebola virus. Cell Host Microbe 2018; 24:221–33 e5. 30092199 10.1016/j.chom.2018.07.009 PMC6298217

[3] Saphire EO, Schendel SL, Fusco ML, et al. Systematic analysis of monoclonal antibodies against Ebola virus GP defines features that contribute to protection. Cell 2018; 174:938–52.e13. 30096313 10.1016/j.cell.2018.07.033 PMC6102396

[4] Saphire EO, Schendel SL, Gunn BM, Milligan JC, Alter G Antibody-mediated protection against Ebola virus. Nat Immunol 2018; 19:1169–78. 30333617 10.1038/s41590-018-0233-9 PMC6814399

[5] Baize S, Leroy EM, Georges-Courbot MC, et al. Defective humoral responses and extensive intravascular apoptosis are associated with fatal outcome in Ebola virus-infected patients. Nat Med 1999; 5:423–6. 10202932 10.1038/7422

[6] Sakabe S, Sullivan BM, Hartnett JN, et al. Analysis of CD8(+) T cell response during the 2013–2016 Ebola epidemic in West Africa. Proc Nat Acad Sci U S A 2018; 115:E7578–86. 10.1073/pnas.1806200115 PMC6094108 30038008

[7] McElroy AK, Akondy RS, Davis CW, et al. Human Ebola virus infection results in substantial immune activation. Proc Nat Acad Sci U S A 2015; 112:4719–24. 10.1073/pnas.1502619112 PMC4403189 25775592

[8] Bornholdt ZA, Turner HL, Murin CD, et al. Isolation of potent neutralizing antibodies from a survivor of the 2014 Ebola virus outbreak. Science 2016; 351:1078–83. 26912366 10.1126/science.aad5788 PMC4900763

[9] Brown EP, Licht AF, Dugast AS, et al. High-throughput, multiplexed IgG subclassing of antigen-specific antibodies from clinical samples. J Immunol Methods 2012; 386:117–23. 23023091 10.1016/j.jim.2012.09.007 PMC3475184

[10] Sholukh AM, Watkins JD, Vyas HK, et al. Defense-in-depth by mucosally administered anti-HIV dimeric IgA2 and systemic IgG1 mAbs: complete protection of rhesus monkeys from mucosal SHIV challenge. Vaccine 2015; 33:2086–95. 25769884 10.1016/j.vaccine.2015.02.020 PMC4411954

[11] Bramble MS, Hoff N, Gilchuk P, et al. Pan-filovirus serum neutralizing antibodies in a subset of Congolese Ebolavirus infection survivors. J Infect Dis 2018; 218:1929–36. 30107445 10.1093/infdis/jiy453 PMC6217721

[12] Sips M, Krykbaeva M, Diefenbach TJ, et al. Fc receptor-mediated phagocytosis in tissues as a potent mechanism for preventive and therapeutic HIV vaccine strategies. Mucosal Immunol 2016; 9:1584–95. 26883728 10.1038/mi.2016.12 PMC4988947

[13] Khurana S, Fuentes S, Coyle EM, Ravichandran S, Davey RT Jr, Beigel JH Human antibody repertoire after VSV-Ebola vaccination identifies novel targets and virus-neutralizing IgM antibodies. Nat Med 2016; 22:1439–47. 27798615 10.1038/nm.4201

