# Supplementary figures and images for: Survivors of Ebola Virus Disease Develop Polyfunctional Antibody Responses
Source: J Infect Dis. 2019 Jul 12;221(1):156–61. doi: 10.1093/infdis/jiz364 (PMC7184900; doi:10.1093/infdis/jiz364)

**A.**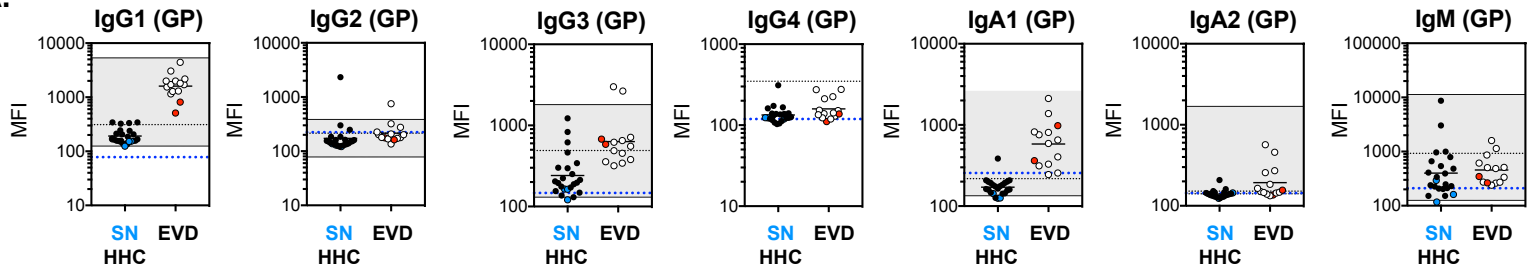**B.**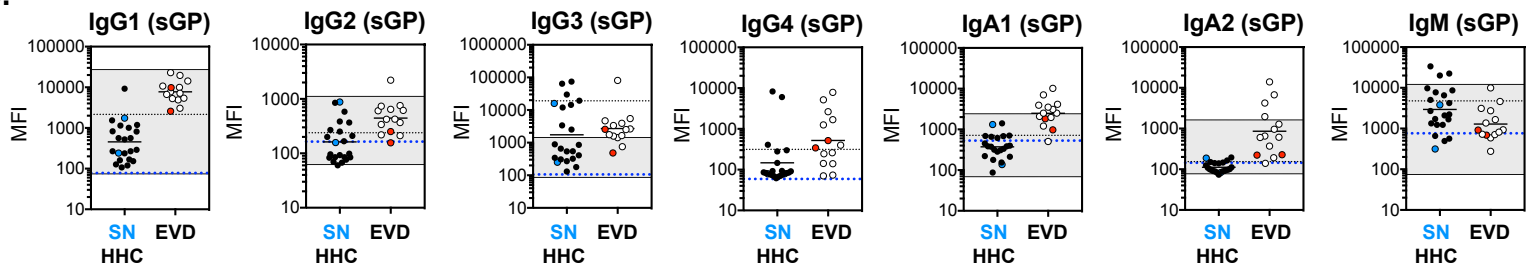**C.**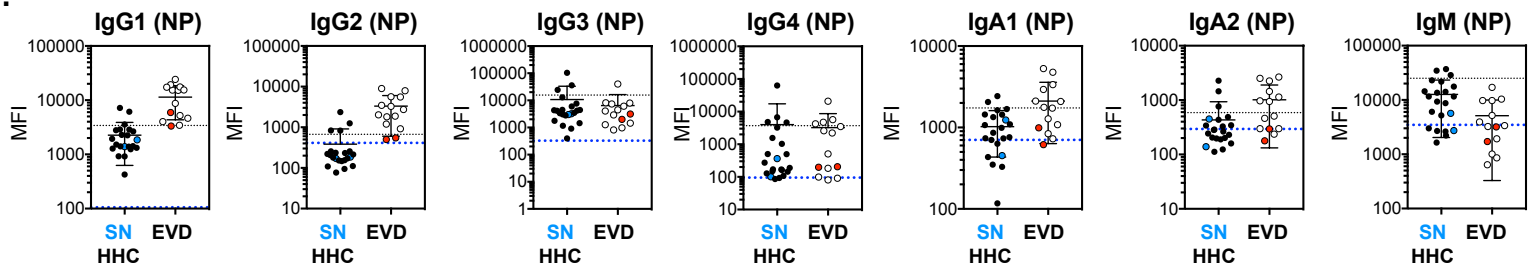**D.**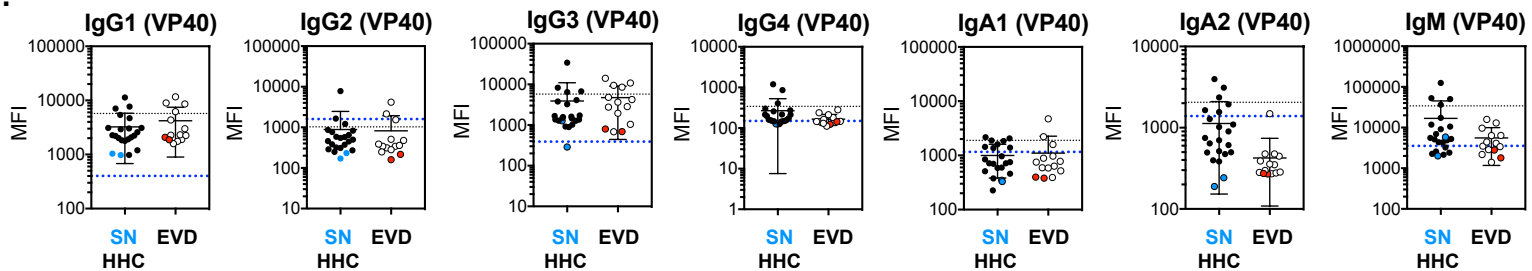

Supplement: jiz364_suppl_Supplementary_Figure_S1 [file jiz364_suppl_supplementary_figure_s1.pdf]

Figure S2

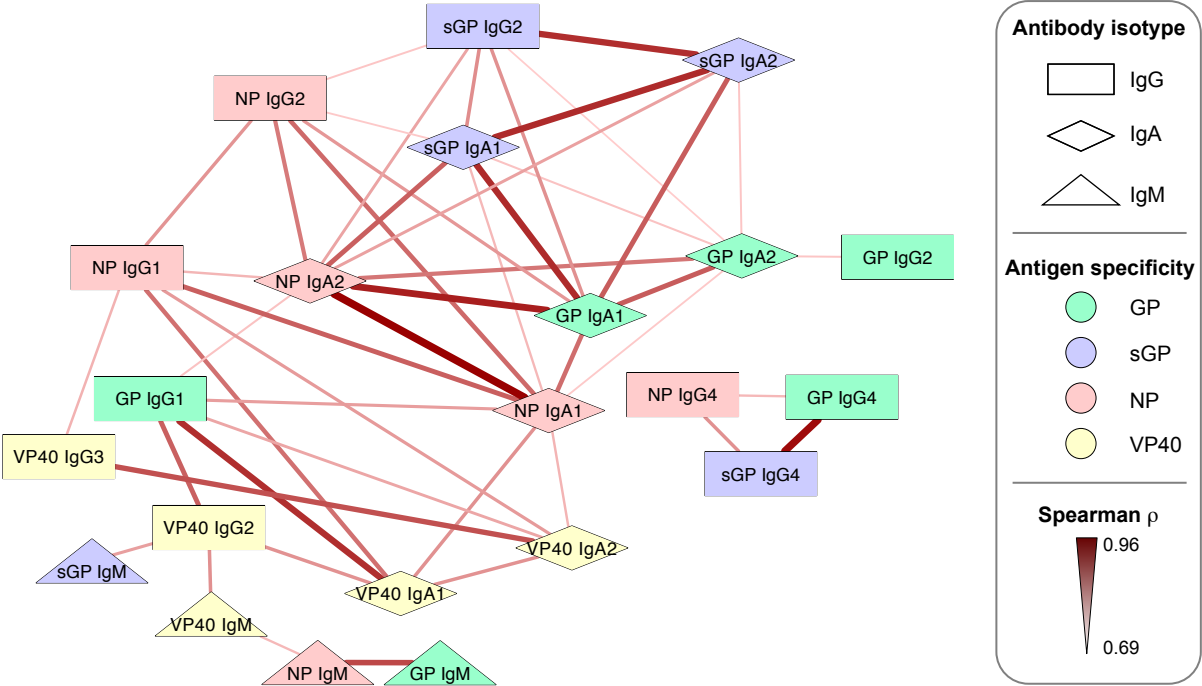

Supplement: jiz364_suppl_Supplementary_Figure_S2 [file jiz364_suppl_supplementary_figure_s2.pdf]
