# Supplementary material for: Survivors of Ebola Virus Disease Develop Polyfunctional Antibody Responses
Source: J Infect Dis. 2019 Jul 12;221(1):156–61. doi: 10.1093/infdis/jiz364 (PMC7184900; doi:10.1093/infdis/jiz364)
Supplement: jiz364_suppl_Supplementary_Legends [file jiz364_suppl_supplementary_legends.docx]

**Figure S1. Antigen-specific antibody responses in EVD survivors.**

Antigen-specific antibody levels in seronegative individuals (SN; blue circles), household contacts (HHC; closed circles) or EVD survivors (open circles) were determined by multiplexed bead analysis for EBOV GP **(A)**, sGP **(B)**, NP **(C)**, or VP40 **(D)**. EVD survivors with low induction of antibody effector function are indicated by the red circles. The shaded box represents the range of a standard curve of GP and sGP-specific polyclonal antibodies. The thin dashed line indicates the cutoff for antibody reactivity against a given antigen and was calculated based on 2X the standard deviation and the geometric mean of the seronegative and household contact group. The thick blue dotted line represents the level of binding observed in a plasma sample from a healthy individual from the Boston, Massachusetts area with no known exposure to Ebola virus.

**Figure S2. Development of IgG1 and IgA responses in EVD survivors is coordinated across EBOV antigen specificities.**

Correlation network analysis of statistically significant associations (Bonferroni adjusted p-value <0.05) between GP-, sGP-, NP-, and VP40-specific antibody levels. Positive correlations between features are indicated by a red connecting line, and the strength of correlation is indicated by weight of the connecting line, and shape of each node indicates the antibody isotype (e.g. IgG, IgA, IgM), and the background color of each node indicates the antigen-specificity (e.g. GP, sGP, VP40, NP), as indicated in the boxed legend.

**Table S1.** **Patient identification codes.**

The manuscript identification code (Patient ID), the patient public identification code (Public ID), and the patient group (EVD survivor, household contact, or seronegative individuals) are shown.
