# Supplementary material for: Survivors of Ebola Virus Disease Develop Polyfunctional Antibody Responses
Source: J Infect Dis. 2019 Jul 12;221(1):156–61. doi: 10.1093/infdis/jiz364 (PMC7184900; doi:10.1093/infdis/jiz364)
Supplement: jiz364_suppl_Supplementary_Table_S1 [file jiz364_suppl_supplementary_table_s1.docx]

| **Patient ID** | **Public ID** | **Group** |
| --- | --- | --- |
| EVD-01 | S-6247670 | EVD survivor; 6 months from acute EVD infection |
| EVD-02 | S-1195170 | EVD survivor; 6 months from acute EVD infection |
| EVD-03 | S-1740090 | EVD survivor; 6 months from acute EVD infection |
| EVD-04 | S-6827870 | EVD survivor; 6 months from acute EVD infection |
| EVD-05 | S-5792800 | EVD survivor; 6 months from acute EVD infection |
| EVD-06 | S-5987500 | EVD survivor; 6 months from acute EVD infection |
| EVD-07 | S-0021700 | EVD survivor; 6 months from acute EVD infection |
| EVD-08 | S-0219160 | EVD survivor; 6 months from acute EVD infection |
| EVD-09 | S-0937800 | EVD survivor; 6 months from acute EVD infection |
| EVD-10 | S-2193700 | EVD survivor; 6 months from acute EVD infection |
| EVD-11 | S-8158490 | EVD survivor; 6 months from acute EVD infection |
| EVD-12 | 14-222232 | EVD survivor; 6 months from acute EVD infection |
| EVD-13 | 14-057241 | EVD survivor; 6 months from acute EVD infection |
| EVD-14 | 14-821913 | EVD survivor; 6 months from acute EVD infection |
| HH-01 | C-0021703 | Household contact |
| HH-02 | C-6015411 | Household contact |
| HH-03 | C-2827732 | Household contact |
| HH-04 | C-6827871 | Household contact |
| HH-05 | C-6809171 | Household contact |
| HH-06 | C-6394111 | Household contact |
| HH-07 | C-7676062 | Household contact |
| HH-08 | C-8870442 | Household contact |
| HH-09 | C-5840773 | Household contact |
| HH-10 | C-9126842 | Household contact |
| HH-11 | C-0546233 | Household contact |
| HH-12 | C-5392022 | Household contact |
| HH-13 | C-4279152 | Household contact |
| HH-14 | C-0030081 | Household contact |
| HH-15 | C-3200972 | Household contact |
| HH-16 | C-1529691 | Household contact |
| HH-17 | C-2218441 | Household contact |
| HH-18 | C-4069722 | Household contact |
| HH-19 | C-1981552 | Household contact |
| HH-20 | C-8743183 | Household contact |
| SN-01 | N/A | Seronegative |
| SN-02 | N/A | Seronegative |
| SN-03 | G13-015029 | Seronegative |
